# Biological function and research progress of *N* ^6^-methyladenosine binding protein heterogeneous nuclear ribonucleoprotein A2B1 in human cancers

**DOI:** 10.3389/fonc.2023.1229168

**Published:** 2023-07-20

**Authors:** Yue Wu, An Li, Can Chen, Zhang Fang, Lujun Chen, Xiao Zheng

**Affiliations:** ^1^ Department of Tumor Biological Treatment, The Third Affiliated Hospital of Soochow University, Changzhou, Jiangsu, China; ^2^ Jiangsu Engineering Research Center for Tumor Immunotherapy, The Third Affiliated Hospital of Soochow University, Changzhou, Jiangsu, China; ^3^ Institute for Cell Therapy, Soochow University, Changzhou, Jiangsu, China

**Keywords:** epigenetics, m^6^A, hnRNPA2B1, tumor, biological mechanisms, cancer therapy

## Abstract

N^6^-methyladenosine (m^6^A) is the most prevalent internal modification found in both mRNA and lncRNA. It exerts reversible regulation over RNA function and affects RNA processing and metabolism in various diseases, especially tumors. The m^6^A binding protein, hnRNPA2B1, is extensively studied as a member of the heterogeneous nuclear ribonucleoprotein (hnRNP) protein family. It is frequently dysregulated and holds significant importance in multiple types of tumors. By recognizing m^6^A sites for variable splicing, maintaining RNA stability, and regulating translation and transport, hnRNPA2B1 plays a vital role in various aspects of tumor development, metabolism, and regulation of the immune microenvironment. In this review, we summarized the latest research on the functional roles and underlying molecular mechanisms of hnRNPA2B1. Moreover, we discussed its potential as a target for tumor therapy.

## 1 Introduction

### 1.1 Epigenetics and m^6^A

Epigenetics is now widely recognized as the study of stable and heritable alterations that occur in chromosomal regions without changes in the DNA sequence (1). Regulatory mechanisms of epigenetic modifications include DNA methylation, histone modification, noncoding RNA regulation, chromatin remodeling, and nucleosome positioning (2). Among these mechanisms, RNA methylation modification, specifically N^6^-methyladenosine (m^6^A), holds paramount importance. m^6^A is the most abundant form of internal modification found in mRNA and lncRNA, and it has been shown to play a crucial role in various biological functions (3). In coding transcripts, m^6^A sites are predominantly located near the 3’-untranslated terminal region (UTR) (4) and translated near the 5’-UTR (5), while they can be found anywhere in noncoding transcripts. These modifications affect RNA alternative splicing (6), stability maintenance (7, 8), and regulation of translation and transport (4, 9). Regulators involved in m^6^A modifications can be categorized into three types: the multicomponent m^6^A methyltransferase complex (MTC) (10, 11), RNA demethylases (12, 13), and m^6^A binding proteins (14, 15). The multicomponent m^6^A methyltransferase complex, comprising METTL3, METTL14, RBM15, WTAP, and KIAA1429, is commonly referred to as the “writers” as it adds m^6^A modifications to target RNA (16). Conversely, the RNA demethylases FTO and ALKBH5, known as “erasers”, are responsible for removing m^6^A modifications (17, 18). These m^6^A sites can be recognized by various m^6^A binding proteins categorized as “readers”, including the IGF2BP proteins, the YTH domain family of proteins and the HNRNP protein family (19–21). The dynamic regulation of m^6^A modifications has been shown to play a significant role in various processes related to tumor development and drug susceptibility.

An illustrative example is the abnormal overexpression of ALKBH5 in acute myeloid leukemia (AML), which serves as a prognostic indicator for poor outcomes and plays a pivotal role in the self-renewal of cancer stem cells (22). Additionally, in HIF2α ^low/−^ clear cell renal cell carcinoma (ccRCC), FTO displays elevated expression levels, leading to increased sensitivity of ccRCC to BRD9 inhibitors (23).

### 1.2 Protein structure and biological function of hnRNPA2B1

HnRNPs represent a diverse group of RNA-binding proteins that are widely expressed in human tissues and encompass approximately 20 different protein types (24). Among them, hnRNPA2B1 belongs to the A/B subfamily of hnRNPs. The hnRNPA2B1 pre-mRNA exhibits four splice subtypes: B1, A2, B1b, and A2b (25, 26), although B1b and A2b are typically not expressed in human cells. Distinguishing between the isoforms of hnRNPA2B1 has proven challenging due to a mere 12-amino acid difference near the N-terminus between A2 and B1 (27).

**Figure 1:**
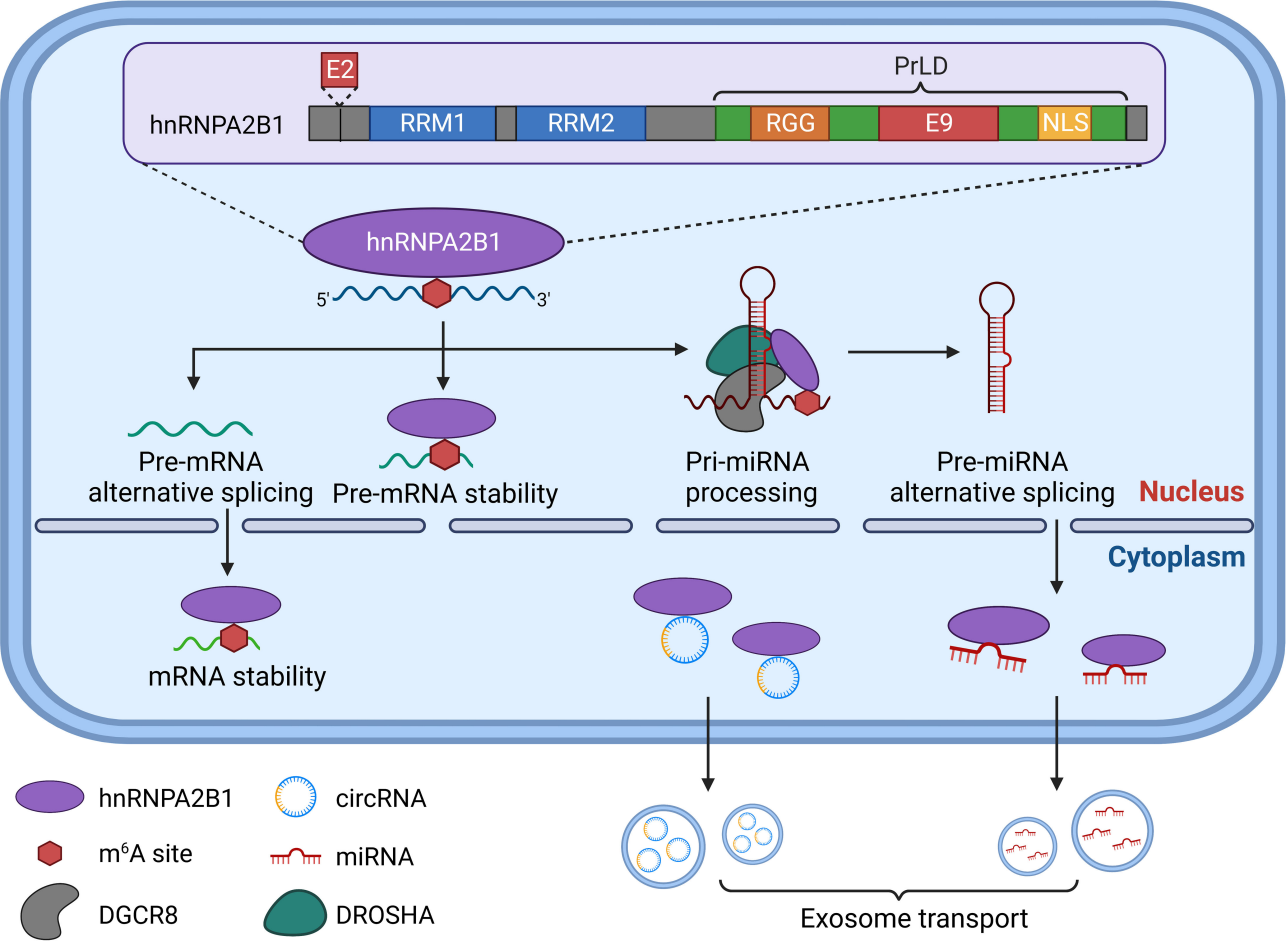
The protein structure and biological function of hnRNPA2B1. RRM1, RRM2 at N-terminal and RGG box, PrLD and LNS at C-terminal constitute the important protein structure of hnRNPA2B1. Differentiating between the A2 and B1 has been challenging due to only a 12-amino acid (Exon 2) near N-termina. For m^6^A modification-dependent functions, hnRNPA2B1 regulates mRNA or miRNA splicing efficiency by binding m^6^A-containing pre-mRNA or pre-miRNA. In addition, hnRNPA2B1 regulates mRNA or pre-mRNA stability as a trans-acting RNA-binding protein. HnRNPA2B1 also specifically recognizes and binds to pri-miRNA-containing transcripts and enhances the binding of DGCR8 to pri-miRNA transcripts, promoting pri-miRNA processing. Moreover, hnRNPA2B1 can load circRNA and miRNA into exosomes to mediate their transport.

Structurally, hnRNPA2B1 consists of two RNA recognition motifs (RRMs) located at its N-terminus, referred explicitly to as RRM1 and RRM2. Additionally, it contains a glycine-rich low-complexity region (LC) at its C-terminus, which encompasses an RGG box, a core PrLD, and an M9-NLS (28–30) (**Figure 1**). The RRMs and RGG box are crucial in RNA binding (24, 30). HnRNPA2B1 interacts with specific RNA sequences and is involved in m^6^A modification through its RRMs (31). While the RGG box significantly influences binding strength, its impact on specific RNA binding is relatively minor (32).

Presently, two main perspectives exist regarding the various mechanisms by which hnRNPA2B1 contributes to m^6^A modification regulation. Alarcón et al. have demonstrated that hnRNPA2B1 binds to RNA sites containing the RGAC motif in nuclear transcripts, thereby promoting m^6^A-dependent pre-miRNA processing events (33). However, another study suggests that hnRNPA2B1 may not directly act on the m^6^A locus. Instead, it may interact with specific targets containing AGG and UAG motifs to activate an “m^6^A switch” by binding to the RRM1 and RRM2 domains (30). Through the utilization of m^6^A markers, hnRNPA2B1 participates in nearly every step of RNA synthesis and processing. It specifically recognizes and binds to transcripts containing pri-miRNAs, enhancing the binding of DGCR8 to pri-miRNA transcripts and facilitating pri-miRNA processing (33). Recent research has also identified a role for hnRNPA2B1 in exosome loading, where sumoylated hnRNPA2B1 can bind and sort miRNAs into exosomes (34). Furthermore, hnRNPA2B1 is involved in regulating mRNA splicing efficiency, potentially through its binding to m^6^A-containing pre-mRNA (35). Additionally, as a trans-acting RNA-binding protein, hnRNPA2B1 contributes to the regulation of mRNA stability (36).

In addition to affecting RNA methylation modification, hnRNPA2B1 can also change its metabolism through ubiquitination, acetylation, and other post-translational modifications. For example, in liver cancer cells, lncRNA miR503HG promotes the degradation of hnRNPA2B1 through ubiquitin-proteasome pathway (37). HnRNPA2B1 also can be acetylated by p300, a common transcriptional coactivator, and promotes nonsmall cell lung cancer (NSCLC) growth by activating COX-2 signaling (38). Numerous studies have highlighted the frequent dysregulation of hnRNPA2B1 and its crucial role in the biological processes of tumorigenesis and development.

In this review, we focused on recent researches regarding the biological behaviors and underlying mechanisms of hnRNPA2B1 in various aspects, including epithelial-mesenchymal transition (EMT), tumorigenesis, tumor microenvironment (TME), and tumor metabolism. Furthermore, we investigated the potential of targeting hnRNPA2B1 as a therapeutic strategy for tumors.

## 2 HnRNPA2B1 in tumorigenesis and development

Tumor growth arises from uncontrolled cell proliferation and invasion, destroying surrounding tissue and distant metastasis. The process of tumor formation and progression is influenced by mutual dynamic crosstalk between the genomic/epigenetic features of tumor cells and tumor microenvironment (TME) components. Extensive studies have demonstrated that hnRNPA2B1 is upregulated in multiple tumors and is associated with poor prognosis (37, 39, 40). It actively regulates RNA processing, translation, transport, and metabolism, thereby influencing EMT, tumorigenesis, TME, and tumor metabolism. (**Table 1**).

**Table 1:** The role of hnRNPA2B1 in multiple cancers.

Cancer	HnRNPA2B1 level	m^6^A site	Targets	Regulators	Mechanism	Function	Refs
NSCLC	N/A	N/A	Snai1 and Twist	N/A	N/A	EMT	(41)
	N/A	The 1,000–1,500 sequence of LINC01234	MiR-106b-5p	LncRNA LINC01234	Pri-miRNA alternative splicing	Proliferation	(42)
	Up	UUAGG motif in the 3’-UTR	CDK6	MiR-506	mRNA translation	Proliferation	(40)
PC	N/A	N/A	GLUT1 and PKM2	N/A	N/A	Proliferation and metabolic shift from mtOXPHOS to the glycolytic pathway	(43)
HCC	Down	N/A	p52 and p65	miR503HG	mRNA stabilization	Suppress invasion and metastasis	(37)
	Up	N/A	A-Raf	N/A	mRNA alternative splicing	Invasion, apoptosis, proliferation, and resistance to MEK1 inhibition	(44)
GC	N/A	N/A	RPRD1B	LncRNA NEAT1	mRNA stabilization	Fatty acid metabolism and primary tumor lymph node implantation	(45)
CRC	N/A	N/A	Raf-1	LncRNA H19	mRNA stabilization	Migration, invasion and EMT	(46)
	N/A	N/A	Siah1 and Fbxo45	LncRNA RP11	mRNA stabilization	Migration and EMT	(47)
	N/A	3’-UTR of p53	p53	CircMYH9	Pre-mRNA stabilization	Proliferation and serine metabolism	(48)
	Up	The +2133 site in the 3′-UTR near the stop codon of TCF7L2	TCF7L2	LncRNA MIR100HG	mRNA stabilization	Cetuximab resistance, migration and invasion	(39)
BC	Down	UAGGG locus in the 3′-UTR	PFN2	N/A	mRNA stabilization	Migration and invasion	(49)
	N/A	3′-UTR of Bcl-x (exon 3)	Bcl-x	LncRNA BC200	Pre-mRNA alternative splicing	Estrogen resistance	(35)
OC	Up	N/A	Lin28B	N/A	mRNA stabilization	Migration and invasion	(50)
ccRCC	N/A	Pentamer-5 and pentamer-8 of VHLα	VHLα and PKM	N/A	Pre-mRNA alternative splicing	Proliferation, migration, invasion and xenograft tumor growth	(51, 52)
MM	Up	N/A	MiR-92a-2-5p and miR-373-3p	N/A	Pri-miRNA processing	Osteoclast formation and osteogenesis inhibition	(53)
	Up	N/A	ILF3	N/A	mRNA stabilization	Proliferation	(54)
CML	Up	N/A	PYK2	N/A	Pre-mRNA alternative splicing	CML pathogenesis	(55)

NSCLC, nonsmall cell lung cancer; PC, pancreatic cancer; HCC, hepatocellular carcinoma; GC, gastric cancer; CRC, colorectal cancer; BC, breast cancer; OC, ovarian cancer; ccRCC, clear cell renal cell carcinoma; MM, multiple myeloma; CML, chronic myelogenous leukemia.N/A, Not Applicable.

### 2.1 HnRNPA2B1 and EMT

EMT is a biological process where polarized epithelial cells downregulate epithelial characteristics and acquire a mesenchymal phenotype (56). In the context of early lung cancer detection, hnRNPA2B1 has been identified as an early marker of EMT and carcinogenesis using monoclonal antibodies 703D4 (57, 58). The downregulation of E-cadherin and upregulation of vimentin and N-cadherin are early features of EMT initiation (59). Transcription factors, Twist, Snail, Zeb1 and so on, play crucial roles in EMT across various types of cancer (60, 61). In colorectal cancer (CRC), lncRNA H19 binds directly to hnRNPA2B1 and activates Raf/ERK/Snail signaling (46). Another lncRNA RP11 is reported to regulate EMT of CRC as well. It post-translationally degrades Siah1 and Fbxo45 mRNA by binding with hnRNPA2B1, resulting in increased expression of Zeb1 (47). Consistent with this, silencing hnRNPA2B1 can down-regulate the expression of E-cadherin inhibitors, Snai1 and Twist (41). Cancer drug resistance can occur due to the inactivation of chemotherapeutic drugs, altered drug targets, and increased efflux, resulting in reduced drug absorption by cancer cells (62). This complex phenomenon may arise from various mechanisms, such as inhibition of cell death, EMT, cellular heterogeneity, and epigenetic influences (63). In cetuximab resistant CRC, hnRNPA2B1 binds to lncRNA MIR100HG through the RRM2 domain and stabilizes TCF7L2 mRNA, enhancing Wnt/β-catenin signaling (39). The Wnt pathway plays a critical role in regulating EMT. In the context of this study, the Wnt target genes ZEB1 and Slug were found to be significantly regulated when hnRNPA2B1 was overexpressed or knocked down (39). Thus, hnRNPA2B1 served as an essential mediator between lncRNA and its target RNA within the EMT signaling pathway.

### 2.2 HnRNPA2B1 in tumor proliferation, apoptosis, invasion, and metastasis

Alternative splicing (AS) has the capacity to influence mRNA translation, localization, and regulation of stability. As a consequence, it leads to the generation of distinct protein isoforms that exhibit divergent functions, localizations, and varying degrees of relevance in cellular processes (64). HnRNPA2B1 is known to generate multiple proteins with distinct exon combinations and functions by alternative splicing of a single target pre-mRNA (31). The alternative splicing of pre-mRNA by hnRNPA2B1 may contribute to the pathogenesis of chronic myeloid leukemia (CML). With modifying the splicing of the β1-integrin-responsive non-receptor tyrosine kinase (PYK2) mRNA precursor, the expression of full-length Pyk2 increases in BCR/ABL-containing HSCs (55). Bcl-x is a well-known player in apoptosis, and its AS gives rise to two distinct isoforms: the anti-apoptotic Bcl-xL and the pro-apoptotic Bcl-xS, which exert opposing effects on apoptosis (65). Notably, hnRNPA2B1 competes with Sam68 for binding to the Bcl-x pre-mRNA, while also interacting with the lncRNA BC200. The upregulation of Bcl-xL expression enhances uncontrolled cell proliferation in estrogen-dependent breast cancer, a phenomenon that can be reversed by the knockdown of hnRNPA2B1 (35).

Another important RNA-binding protein, DGCR8, forms a complex with the RNase III enzyme Drosha, resulting in the formation of a microprocessor complex responsible for cleaving primary microRNA transcripts (pri-miRs) (66). In NSCLC, hnRNPA2B1 is regulated by the lncRNA LINC01234 and binds to DGCR8 to participate in the splicing of the miR-106b-5p precursor. Mature miR-106b-5p downregulates CRY2 and upregulates c-Myc. Activated c-MYC, in turn, increases the transcription of LINC01234 and promotes proliferation (42). This mechanism is also observed in multiple myeloma (MM) osteolytic bone disease, where the hnRNPA2B1-DGCR8 complex plays a role in pri-miRNA processing events through m^6^A modification. Under this mechanism, the generation of miR-92a-2-5p and miR-373-3p is increased, and they are packaged into exosomes and delivered to monocytes or mesenchymal stem cells (MSCs), activating osteoclast formation and inhibiting osteogenesis by suppressing IRF8 or RUNX2 (53). In the study conducted by Yin et al., it has been demonstrated that hnRNPA2B1 plays a role in the upregulation and modulation of cell division protein kinase 6 (CDK6) expression in NSCLC cell lines. CDK6 is a cyclin-dependent kinase active during interphase, regulating the transition from the G1 to S phase of the cell cycle. Aberrant CDK6 activity is frequently observed in various human cancers, contributing to their development and progression (67). Mechanistically, hnRNPA2B1 recruits the RNA helicase DHX9 and binds to the UUAGG motif in the CDK6 3’-UTR, collaborating with miR-506 to suppress the translation of CDK6 (40).

The transcription and degradation of mRNA are dependent on changes in its concentration (68). mRNA stability is determined by mRNA cis-elements and corresponding trans-acting binding proteins (36). Jiang et al. have revealed that hnRNPA2B1 identifies the m^6^A site of ILF3 mRNA in MM and reduces its fragility, thereby mediating AKT3 to promote MM cell proliferation (54). During migration and invasion in ovarian cancer, hnRNPA2B1 plays a positive regulatory role by interacting with Lin28B mRNA and reducing its degradation (50). However, contrary to the findings in other cancer studies, hnRNPA2B1 shows an inverse correlation with breast cancer (BC) metastasis, and patients with high HNRNPA2B1 expression exhibit better survival and prognosis (49). Based on research findings, hnRNPA2B1 directly binds to the UAGGG region in the 3′-UTR, leading to the degradation of PFN2 mRNA and suppressing the pro-invasive phenotype (49). Consistently, in hepatocellular carcinoma (HCC) cells, hnRNPA2B1 is degraded by miR503HG specifical interaction, which promotes the metabolism of p52 and p65 mRNA, inhibits the NF-κB signaling pathway, and thereby suppresses HCC metastasis (37).

In a word, during the proliferation, apoptosis, invasion, and metastasis of a tumor, hnRNPA2B1 can directly regulate the alternative splicing of pre-mRNA and mRNA stability or cooperate with miRNA on mRNA to regulate downstream signaling.

### 2.3 HnRNPA2B1 and tumor metabolism

Metabolic remodeling plays a crucial role in tumor initiation and progression (69). Major metabolic changes in cancer include increased aerobic glycolysis (70), dysregulated lipid metabolism (71), elevated reactive oxygen species (ROS) generation (72), and disturbances in enzyme activities (73). The ATP-binding cassette transporter (ABCA1) facilitates the formation of high-density lipoprotein (HDL) and enhances plasma HDL levels, thereby promoting cholesterol efflux (74). Cholesterol 7a-hydroxylase (CYP7A1) plays a crucial role in converting cholesterol into bile acids (75). The proper expression of both ABCA1 and CYP7A1 is vital for maintaining cholesterol metabolism homeostasis. In hepatocytic cholesterol metabolism, the intranuclear expression level of hnRNPA2B1 is increased by lncRNA lnc-HC, which forms a protein-RNA complex targeting Cyp7a1 and Abca1, resulting in mRNA destabilization and decreased translation levels (76). SREBP1, a master regulator of fatty acid and triglyceride synthesis, increases lipid accumulation upon activation of c-Jun signaling (77). The lncRNA NEAT1, in conjunction with hnRNPA2B1, targets RPRD1B mRNA stability, activating the c-Jun/c-Fos/SREBP1 axis to promote fatty acid metabolism and primary tumor lymph node implantation in gastric cancer (GC) (45). Mitochondrial uncoupling protein 2 (UCP2) sustains the transition from mitochondrial oxidative phosphorylation (OXPHOS) to aerobic glycolysis, known as the Warburg effect (43). Pyruvate kinase subtype M2 (PKM2) is highly enriched in tumor cells and induces the Warburg effect (78). In PC cells, UCP2 induces hnRNPA2B1 generation through its antioxidant function, resulting in the upregulation of GLUT1 and PKM2 and increased lactate secretion (43). Similarly, in ccRCC, hnRNPA2B1 interacts with VHLα. Through the regulation of pyruvate kinase (PKM) splicing and the subsequent increase in PKM2 expression, hnRNPA2B1 exerts a tumor-suppressive effect on cellular glucose metabolism (51). The negative regulation of hnRNPA2B1 occurs through N-terminal-mediated ubiquitination of VHLα. In contrast, its positive regulation forms a feedback loop that regulates downstream abnormal c-myc signaling, ultimately contributing to its tumor-suppressive effect (52). Activation of oncogenes, such as MYC, transcription factors like HIF1α, and deactivation of tumor suppressor genes like p53 lead to increased glucose uptake and inhibition of OXPHOS (79). In CRC, circMYH9 promotes tumor cell proliferation and serine metabolism. circMYH9 recruits nuclear hnRNPA2B1 to inhibit its combination and stabilization with p53 pre-mRNA. Additionally, an amino acid deficiency increases ROS levels and HIF1α expression, which further enhances circMYH9 expression, forming a positive feedback loop (48). Overall, these results indicate that hnRNPA2B1 covers multiple metabolic imbalances in tumor cells.

### 2.4 HnRNPA2B1 in the TME

**Figure 2:**
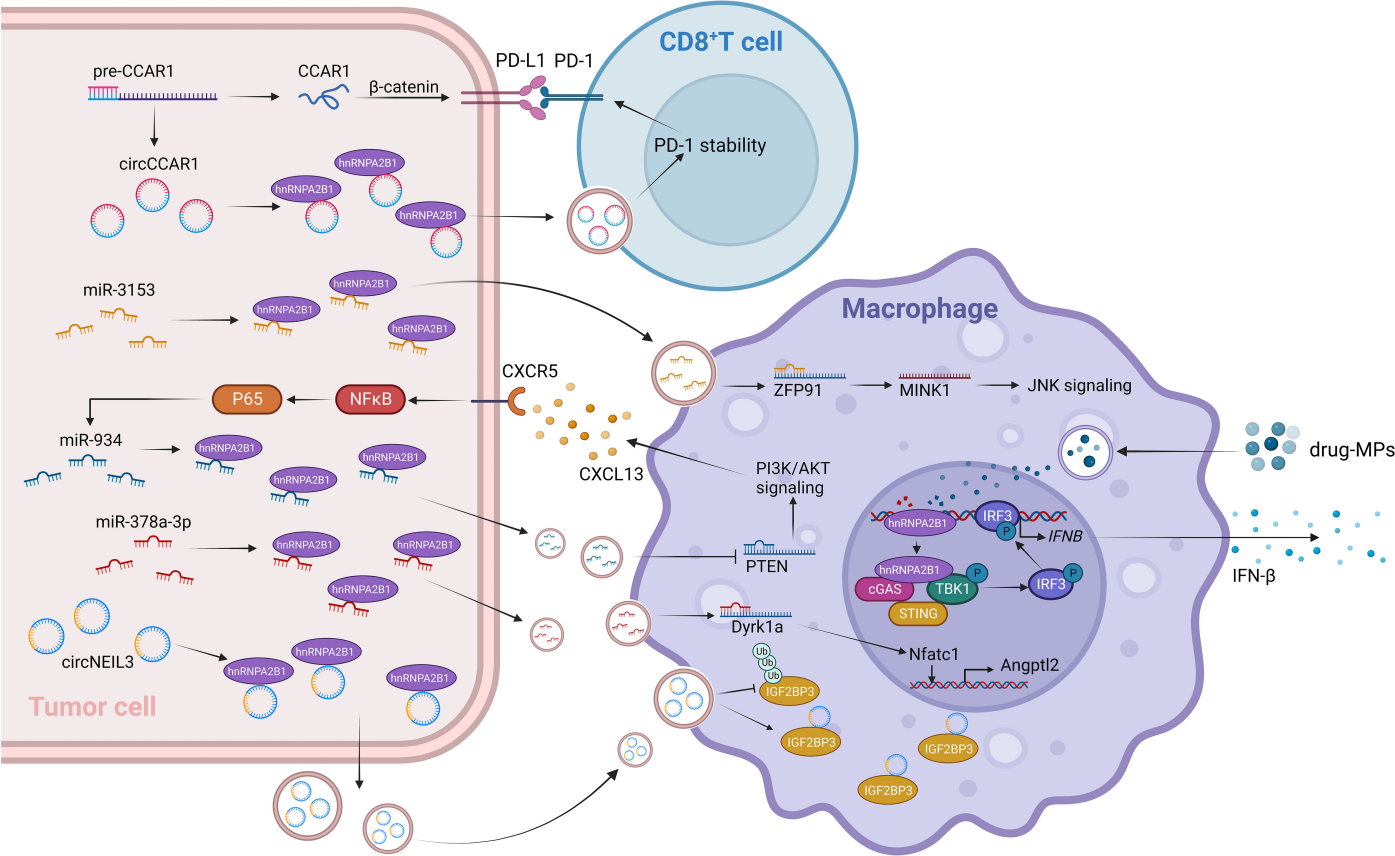
HnRNPA2B1 plays a role in tumor immunity by inhibiting the immune response through the transport of miRNA and circRNA to recipient immune cells. HnRNPA2B1-circNEIL3 exosomes target TAMs and stabilize IGF2BP3, leading to immunosuppressive properties. Moreover, the uptake of hnRNPA2B1-mediated tumor-derived EVs enriched with miR-378a-3p by BMMs activates the Dyrk1a/Nfatc1/Angptl2 axis, promoting prostate cancer bone metastasis. Macrophages exist in two phenotypes: proinflammatory (M1) and anti-inflammatory (M2). JNK signaling and PI3K/AKT signaling pathways are associated with M2 polarization. HnRNPA2B1 mediates the secretion of exosomes containing miR-3153 and miR-934, which respectively activate JNK signaling and PI3K/AKT signaling in macrophages, promoting M2 polarization. Some drugs have been reported to reverse M2 polarization. Drug-induced M1-shifted macrophages increase IFN-β secretion through the hnRNPA2B1-cGAS axis. Furthermore, hnRNPA2B1 contributes to a decrease in the effectiveness of immune checkpoint inhibitors. Exosomal circCCAR1, secreted in a hnRNPA2B1-dependent manner, is taken up by CD8^+^ T cells and stabilizes the interaction between PD-1 and PD-L1, promoting resistance to anti-PD-1 immunotherapy.

Recent research has revealed the significant involvement of the m^6^A family in tumor immunity, drawing extensive attention due to its impact on the effectiveness of immune checkpoint blockade therapy in cancer cells (80, 81). HnRNPA2B1 has been found to be significantly overexpressed in esophageal cancer and associated with the regulation of the TME (82). Overexpression of hnRNPA2B1 leads to the enrichment of tumor-derived extracellular vesicles (EVs) containing miR-378a-3p in prostate cancer bone metastases. Upon uptake by bone marrow macrophages (BMMs), these EVs target and activate the Dyrk1a/Nfatc1/Angptl2 axis, promoting bone metastasis (83). Tumor-associated macrophages (TAMs), consisting of M2 and M1 cells, not only fail to recognize and engulf tumor cells but also contribute to immune evasion (84). Recent research in adrenocortical carcinoma (ACC) has shown that HNRNPA2B1 affects tumor development by regulating infiltration of M0 macrophages into the TME (85). Drug-induced DNA damage in macrophages stimulates the secretion of IFN-β through the hnRNPA2B1-cGAS axis (86). Exosomes, natural carriers known for their role in overcoming chemoresistance, have a significant impact on tumor cell transcriptome changes and immune responses (87). The composition of exosomes, which includes diverse nucleic acids, plays a crucial role in facilitating these processes (88). Elaborate studies have demonstrated that hnRNPA2B1 induces the packaging of circNEIL3 into exosomes, which are then taken up by infiltrating TAMs. This enables the stabilization of oncogenic protein IGF2BP3, conferring immunosuppressive properties and promoting glioma progression (89). Furthermore, hnRNPA2B1 is involved in the polarization of M2 macrophages. In LUAD, hnRNPA2B1 facilitates the delivery of exosomal miR-3153 to activate JNK signaling and induce M2 macrophage polarization (90). Furthermore, in CRC, hnRNPA2B1 binds to the GGAG sequence and facilitates the packaging of miR-934 into exosomes, which are transferred to recipient macrophages. This leads to downregulation of PTEN expression, activation of the PI3K/AKT signaling pathway, and further polarization of macrophages. The resulting M2 macrophages secrete CXCL13, which acts on the CXCR5 receptor, activating downstream NFκB/p65/miR-934 signaling in CRC cells (91). A study of CD8^+^ T cells found that hnRNPA2B1-dependent secretion of exosomal circCCAR1 leads to its uptake by CD8^+^ T cells, causing their dysfunction by stabilizing the expression of PD-1 and promoting resistance to anti-PD-1 immunotherapy (92) (**Figure 2**). The crucial role of hnRNPA2B1-mediated vesicle transport in tumor immunity is evident. Consequently, discovering the novel role of hnRNPA2B1 in the tumor microenvironment opens up new avenues and ideas for future tumor immunotherapy.

## 3 Targeting hnRNPA2B1 in cancer treatment

The therapeutic targeting of hnRNPA2B1 shows promise for cancer treatment due to its significant impact on multiple cancers. A series of drugs targeting different functions of hnRNPA2B1 have emerged.

### 3.1 Current status of drug therapy

**Figure 3:**
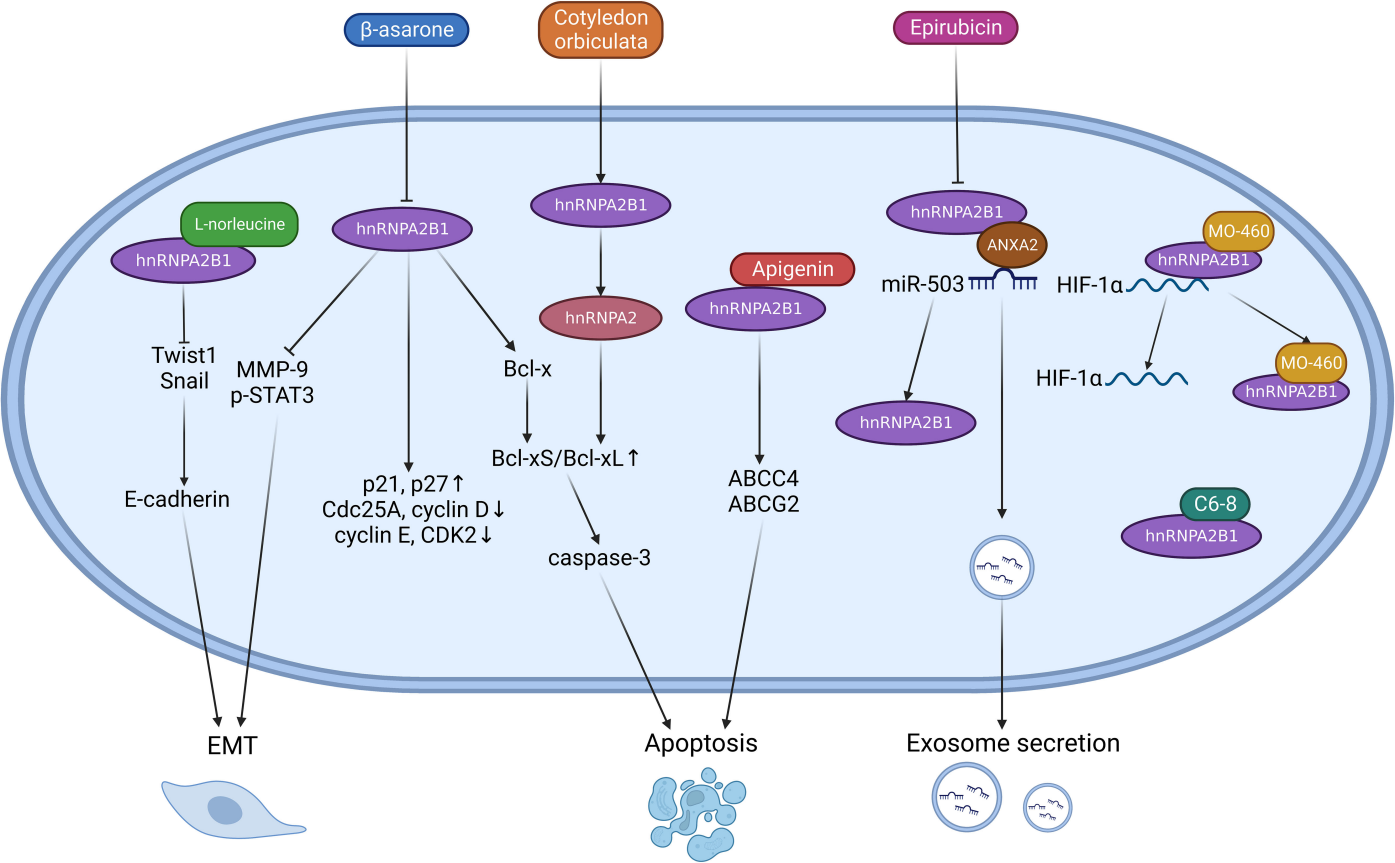
Targeting hnRNPA2B1 for cancer treatment involves the use of certain drugs that act as inhibitors, although the exact mechanisms of action are not fully understood. Inhibition of hnRNPA2B1 by β-asarone leads to reduced expression of MMP-9 and p-STAT3, thereby decreasing EMT. Additionally, β-asarone promotes the splicing of Bcl-xS, which mediates tumor cell apoptosis. Similarly, cotyledon orbiculate induces apoptosis through the same effect. Epirubicin inhibits the interaction between hnRNPA2B1 and the miR503-ANAX2 complex, facilitating exosome transport. Some drugs bind directly to specific regions of hnRNPA2B1. L-norleucine binds to the RRM1 and RRM2 domains of hnRNPA2B1, inhibiting EMT by regulating transcription factors like Twist1 and Snail. Apigenin directly acts on hnRNPA2B1, promoting apoptosis induced by ABCC4 and ABCG2. MO-460 competes with HIF-1α for binding to hnRNPA2B1, leading to reduced translational expression of HIF-1α. C6-8 exert antitumor effects by directly targeting hnRNPA2B1.

In glioma cells (U251), β-asarone inhibits hnRNPA2B1, leading to increased MMP-9 and p-STAT3 expression and the induction of a malignant phenotype (93). β-asarone also promotes the processing of Bcl-x, shifting the balance from Bcl-xL to Bcl-xS, which may contribute to apoptosis through the mitochondrial pathway (94). Extracts from the South African medicinal plant Cotyledon orbiculata cause the splicing of hnRNPA2B1 to the hnRNPA2 subtype, also increasing the ratio of Bcl-xS/Bcl-xL and activating caspase-3 cleavage and apoptosis (95). Epirubicin treatment induces the secretion of exosomes carrying anti-tumoral miR-503 by preventing hnRNPA2B1 from interacting with miR-503/ANXA2 in endothelial cells (96). The natural product MO-460 binds to the glycine-rich domain of HNRNPA2B1, inhibiting its association with the 3′-UTR of HIF-1 (97). The aptamer C6-8, targeting ROS 17/2.8 in rat osteosarcoma cells, inhibits tumor cell growth by specifically binding to hnRNPA2B1 after conjugation with fluorescent carbon nanodots (CDots) (98). L-norleucine interacts with the RRM1 and RRM2 domains of hnRNPA2B1, inhibiting Twist1 and Snail and increasing E-cadherin, thereby hindering EMT and tumor metastasis (99). Dietary flavones like apigenin have also been considered potential chemo-sensitizers. Apigenin sensitizes triple-negative breast cancer (TNBC) spheroids to doxorubicin through its interaction with hnRNPA2B1, promoting apoptosis induced by ABCC4 and ABCG2 transporters (100) (**Figure 3**).

### 3.2 Challenges and future perspectives

As previously mentioned, hnRNPA2B1 exhibits expression in various human tissues, and the potential toxicity associated with targeted therapy utilizing this molecule is evident. Currently, research on hnRNPA2B1 targeted therapy is primarily focused on tumor cells, necessitating further animal experiments to validate the feasibility of this molecular targeted drug.

Furthermore, understanding how hnRNPA2B1 exerts opposing roles in the same cancer type requires careful investigation. It is worth noting that in BC and kidney cancer (49, 101), hnRNPA2B1 plays a tumor-promoting or tumor-suppressing role in different populations, possibly due to variations in research models, microenvironments, and tumor heterogeneity. Comprehensive research is needed to better understand these complexities in order to effectively guide targeted therapy effectively.

Although there are only 12 amino acid differences between hnRNPA2 and B1, studies have shown that they have slightly different recognizable sequences, indicating that hnRNPA2 and B1 have different functions (102). Previous research has demonstrated that hnRNPA2, rather than hnRNPB1, is upregulated in an inflammation-induced mouse liver cancer model. HnRNPA2 activates the Ras/Raf/MAPK/ERK signaling pathway and promotes tumorigenesis through A-Raf splicing (44). Further studies should delve into the functions and mechanisms of various isoforms of hnRNPA2B1 in m^6^A modification and their implications for targeted therapy.

Proliferation, resistance to growth inhibitory factors, evasion of cell death, replicative immortality, induction of angiogenesis, invasion, and metastasis are considered hallmarks of cancer (103). Recent studies have revealed multiple mechanisms through which hnRNPA2B1 affects cancer. These studies primarily focus on cell proliferation, invasion, metastasis, and dysregulation of metabolism (45, 50, 67). However, emerging evidence suggests that hnRNPA2B1 also plays a crucial role in other biological processes, such as immune microenvironment regulation. For example, Cao et al. have found that hnRNPA2B1 can initiate and amplify the innate immune response to DNA viruses (104). Therefore, hnRNPA2B1 may also mediate antitumor immune responses. Further research is needed to explore the effects and mechanisms of hnRNPA2B1 on each cancer biomarker.

In conclusion, hnRNPA2B1 was identified as a modifier of various RNAs that impacted tumorigenesis and development in an m^6^A-dependent manner. This modification occurred regardless of its dependence or independence on other members of the m^6^A methylase family. HnRNPA2B1-targeted therapy is still in its early stages, and ongoing efforts should be directed toward designing and optimizing tumor treatment strategies involving hnRNPA2B1.

## Author contributions

All authors listed have made a substantial, direct, and intellectual contribution to the work and approved it for publication.
